# Resveratrol Decreases Oxidative Stress by Restoring Mitophagy and Improves the Pathophysiology of Dystrophin-Deficient *mdx* Mice

**DOI:** 10.1155/2018/9179270

**Published:** 2018-10-29

**Authors:** Rio Sebori, Atsushi Kuno, Ryusuke Hosoda, Takashi Hayashi, Yoshiyuki Horio

**Affiliations:** Department of Pharmacology, Sapporo Medical University School of Medicine, S1, W 17, Chu-ouku, Sapporo 060-8556, Japan

## Abstract

We previously showed that treatment with resveratrol (3,5,4′-trihydroxy-*trans-*stilbene), an activator of the NAD^+^-dependent deacetylase SIRT1 at 4 g/kg food for 32 weeks, significantly decreased the muscular reactive oxygen species (ROS) levels and ameliorated the pathology of *mdx* mice, an animal model of *Duchenne* muscular dystrophy (DMD). Here, we treated *mdx* mice with various doses of resveratrol (0.04, 0.4, and 4 g/kg food) for 56 weeks and examined the effects on serum creatine kinase levels and physical activities. Because resveratrol promotes autophagy, we also investigated whether autophagy including mitochondrial autophagy (mitophagy) is involved in resveratrol's effects. Autophagy/mitophagy-related genes and autophagic flux were downregulated in the muscle of *mdx* mice, and these phenomena were reversed by resveratrol with significant ROS reduction. Resveratrol at 4 g/kg food reduced the number of immature myofibers containing central nuclei and fine fibers < 400 *μ*m^2^ and increased that of thicker myofibers in the quadriceps, suggesting that resveratrol decreased myofiber wasting and promoted muscular maturation. Accordingly, resveratrol at 0.4 g/kg food reduced the creatine kinase levels to one-third of those in untreated *mdx* mice and significantly increased the animals' physical activities. In C2C12 myoblast cells, resveratrol promoted mitophagy and eliminated mitochondria containing high superoxide levels. The clearance of damaged mitochondria and ROS reduction by resveratrol was completely suppressed by an autophagy inhibitor (chloroquine) and by knocking down *Atg5* or *Pink1*, essential genes for autophagy and mitophagy, respectively. Thus, resveratrol is a potential therapeutic agent for DMD, and the clearance of damaged mitochondria probably contributes to its action.

## 1. Introduction

Duchenne muscular dystrophy (DMD) is a severe type of muscular dystrophy, in which mutations of the dystrophin gene lead to progressive muscle wasting and degeneration [1]. Few treatments exist for DMD except for glucocorticoids, which increase muscle strength and functional measures in the short term, although their ability to extend walking ability for more than two years is unclear [2]. Furthermore, the long-term glucocorticoid use may cause prediabetes and osteoporosis, and glucocorticoids are not thought to improve myogenesis or fibrosis [2, 3].

SIRT1, an NAD^+^-dependent protein deacetylase, regulates transcription machineries and plays pivotal roles in controlling metabolism, inflammation, differentiation, and DNA repair [4]. SIRT1 promotes cell survival by reducing oxidative stress and by increasing mitochondrial biogenesis by deacetylating and activating Forkhead Box O transcription factors (FOXOs) and peroxisome proliferator-activated receptor gamma coactivator 1-alpha (PGC-1*α*) [4]. Resveratrol (3,5,4′-trihydroxy-*trans*-stilbene), a natural polyphenol found in grapes and red wine, is an activator of SIRT1 [5]. We found that resveratrol at a dose of 4 g/kg food ameliorates the skeletal muscle and cardiac pathologies of dystrophin-deficient *mdx* mice [6, 7]. Beneficial effects of resveratrol in *mdx* mice have also been reported by other groups [8–10], and SIRT1 overexpression in *mdx* mice was shown to reduce muscle damage and improve function [11].

In the heart, resveratrol induces reactive oxygen species- (ROS-) detoxifying enzyme superoxide dismutase 2 (SOD2) by activating nuclear SIRT1, thereby decreasing oxidative damage [12]. Resveratrol also inhibits myocardial hypertrophy and fibrosis by promoting SIRT1's deacetylation of coactivator p300, which then undergoes ubiquitin-dependent degradation [7].

Surprisingly, SOD2 levels in the skeletal muscle of *mdx* mice were not significantly elevated by resveratrol, possibly because SIRT1 was not concentrated in the nuclei of myofibers [6]. Although resveratrol suppressed the upregulation of NADPH oxidase subunits, resveratrol and SIRT1 may use another mechanism to reduce ROS levels in the muscle of *mdx* mice.

Autophagy is a process that digests unnecessary or dysfunctional components in cells. SIRT1 promotes autophagy by deacetylating and activating autophagic components such as Atg5, Atg7, and LC3 [13, 14], and resveratrol induces autophagy by activating cytoplasmic SIRT1 [15, 16]. Damaged or dysfunctional mitochondria, the major source of ROS in most cells [17], are eliminated by an autophagic process called mitophagy [18–20]. The loss of membrane potential in damaged mitochondria causes PTEN-induced putative kinase 1 (Pink1) to accumulate on their outer membrane, where Pink1 recruits, phosphorylates, and activates parkin, a ubiquitin ligase. The activated parkin recruits p62, an autophagy adaptor protein, to the damaged mitochondria, leading to encapsulation of the damaged mitochondria by LC3 in autophagosomes; the mitochondria are then degraded in lysosomes [18–20]. Autophagy insufficiency induced by the knockout of autophagy/mitophagy-related genes such as *Atg3*, *Atg5*, *Atg7*, *LC3B*, and *Pink1* causes a significant increase in cellular ROS [19], suggesting that ROS are liberated from damaged mitochondria that escape mitophagy. In addition, the muscle-specific knockout of *Atg5* or *Atg7* results in muscle atrophy, dysfunction, and myopathy [21, 22]. Notably, mitochondria in the muscle of *Atg7*-null mice are morphologically and functionally abnormal [23]. Loss-of-function mutants of *Pink1* or *parkin*, mitophagy-related genes, show mitochondrial dysfunction and flight muscle degeneration in *Drosophila* [24, 25]. Thus, mitophagy may have a role in the pathology of muscular dystrophies.

Here, we examined autophagy/mitophagy in *mdx* mice and in C2C12 myoblast cells. Because resveratrol can act as a mitochondrial depolarizing agent [26], and because a low dose of resveratrol (2.5 mg/kg/day) improves insulin resistance in mice [27], we investigated the effects of lower doses of resveratrol, i.e., 0.04 and 0.4 g/kg food, as well as 4 g/kg food, on *mdx* mice. Resveratrol increased the expression of autophagy/mitophagy-related genes and autophagic flux and reduced ROS levels in the muscle of *mdx* mice. Furthermore, resveratrol improved the muscular pathology and physical strength of the *mdx* mice. We further showed that mitophagy was indispensable for the ROS reduction caused by resveratrol in C2C12 myoblast cells.

## 2. Materials and Methods

### 2.1. Reagents and Antibodies

Resveratrol (185-01721) and Hoechst 33342 (346-07951) were from Wako Pure Chemicals (Osaka, Japan). Food grade resveratrol for mouse treatment was from ChromaDex (ASB-00018089-101, Irvine, CA). FITC-conjugated wheat germ agglutinin (WGA) lectin (W834), dihydroethidium (DHE) (D1168), MitoSOX Red (M36008), MitoTracker Red (MTR, M7512), and Lipofectamine RNAiMAX Transfection Reagent (13778-150) were from Thermo Fisher Scientific (Rockford, IL). The RNeasy Fibrous Tissue Mini Kit (74704) was from Qiagen (Valencia, CA). The GoScript Reverse Transcription System (A6010), GoTaq qPCR Master Mix (A600A), and ViaFect Transfection Reagent (E4982) were from Promega (Madison, WI). Antimycin A (A8674) and chloroquine (CQ) (C6628) were from Sigma-Aldrich (St. Louis, MO). Plasmid EGFP-LC3 Expression Vector was from Addgene (#11546). siRNAs against mouse Atg5 (SASI_Mm01_00089196), mouse Pink1 (SASI_Mm02_00331134), and MISSION siRNA Universal Negative Control (SIC-001) were from Sigma Genosys Japan (Ishikari, Japan). Antibodies used were as follows: anti-LC3AB (#12741), anti-phosho-Ser65-4EBP1 (#9451), anti-total 4EBP1 (#9452), and anti-ubiquitin (#3936) from Cell Signaling Technology (Beverly, MA), anti-p62 (GP62-C) from Progen (Heidelberg, Germany), and anti-GAPDH (G8795) and anti-*α*-tubulin (T5168) from Sigma-Aldrich.

### 2.2. Animals and Experimental Design

All *in vivo* experiments were conducted in strict accordance with the Guide for the Care and Use of Laboratory Animals (Institute of Laboratory Animal Resources, 1996) and approved by the Animal Care and Use Committee of Sapporo Medical University. Male C57BL/10ScSn-Dmdmdx/J mice (*mdx* mice) and age-matched C57BL10 mice were purchased from Oriental Yeast Co. Ltd. (Tokyo, Japan). C57BL10 mice served as WT. Muscle tissue samples were prepared at 22 weeks of age. In a series of experiments, the effects of resveratrol were analyzed in 24 *mdx* mice. *mdx* mice were orally given 0, 0.04, 0.4, or 4 g resveratrol/kg food ad libitum from 9 weeks to 65 weeks of age (6 mice for each dose). At 65 weeks of age, the mice were sacrificed and their quadriceps were frozen in liquid nitrogen-cooled isopentane (Nacalai Tesque, Kyoto, Japan) and stored at −80°C until use.

### 2.3. Gene Expression Assay

Total RNA was prepared from quadriceps muscles using the RNeasy Fibrous Tissue Mini Kit. Complementary DNA generated with the GoScript Reverse Transcription System was analyzed by the StepOne Real-Time PCR System (Applied Biosystems, Foster City, CA) using the GoTaq qPCR Master Mix. Each sample was run in duplicate, and the mean value was used to calculate the mRNA level of the gene of interest. All data were normalized to 18s ribosomal RNA using the standard curve method. The primer sequences are listed in Supplemental Table 1.

### 2.4. Western Blotting

Frozen quadriceps muscles were powdered by mortar and pestle, lysed in ice-cold CelLytic M Tissue Lysis Reagent (C3228, Sigma-Aldrich) with a 1% protease inhibitor cocktail (25955-11, Nacalai Tesque, Kyoto, Japan) and 1% phosphatase inhibitor cocktail (07574-61, Nacalai Tesque), and centrifuged at 10,000*g* for 10 min at 4°C. C2C12 samples were homogenized in ice-cold CelLytic M Cell Lysis Reagent (C3228, Sigma-Aldrich) with the above-described protease inhibitor and phosphatase inhibitor cocktails. The protein concentration of the supernatant was measured using the Protein Quantification Kit-Rapid (PQ01, Dojindo, Kumamoto, Japan). Supernatant fractions of equal protein concentration were analyzed by Western blotting as described previously [6].

### 2.5. Histological Analyses

Frozen muscles were embedded in optimal cutting temperature compound (Tissue-Tek, Torrance, CA), and blocks were cross-sectioned mid-belly at 5 *μ*m by cryostat at −20°C. To monitor tissue ROS levels, sections of quadriceps muscles were incubated with 5 *μ*M DHE (Thermo Fisher Scientific) for 30 min at 37°C and washed twice with PBS. The digital images were captured using an inverted confocal laser scanning microscope (LSM510META; Zeiss, Germany) at 512 × 512 pixels, with a 63× oil immersion objective lens. The DHE fluorescence intensity was quantified by the ImageJ software (National Institutes of Health, Bethesda, MD). The fluorescence intensity was measured from 6 randomly selected images of each muscle, and the average of 4 mice in each group was determined.

To analyze cross-sectional areas, sections of quadriceps muscles were labeled with FITC-conjugated WGA. Nuclei were stained with Hoechst 33342. The digital images were captured by an LSM510META inverted confocal laser canning microscope, and the cross-sectional areas and central nuclei were quantified by the ImageJ software. The cross-sectional areas of approximately 300 randomly selected myofibers per muscle were measured. The percentage of fibers with centrally located nuclei was analyzed in 480–500 myofibers per muscle in each group.

### 2.6. Cell Culture

C2C12 myoblast cells were cultured in Dulbecco's modified Eagle's medium (Wako Pure Chemical) supplemented with a 1% antibiotic-antimycotic mixed stock solution (Nacalai Tesque) and 10% fetal bovine serum (MP Biomedicals, Solon, OH).

### 2.7. Transfection of siRNA

Lipofectamine RNAiMAX Transfection Reagent was used to transfect siRNAs (30 nM) targeting *Atg5* and *Pink1*, according to the manufacturer's instructions. Cells were analyzed 48 h after transfection.

### 2.8. Analysis of Mitophagy in C2C12 Cells

C2C12 cells were transfected with EGFP-LC3 using ViaFect Transfection Reagent (Promega) according to the manufacturer's instructions and then were stained with 200 nM MTR 42 h after transfection. The cells were incubated with vehicle or 30 *μ*M resveratrol for 6 h. In some samples, 50 *μ*M CQ was added before the resveratrol treatment. After fixation, the colocalization of EGFP-LC3 with mitochondria was analyzed by confocal laser microscopy. After mitochondria take-up MTR in a membrane potential-dependent manner, the MTR fluorescence is retained, even if the mitochondrial potential is lost during fixation. The number of EGFP-LC3 dots colocalized with MTR was counted in at least 30 randomly selected cells in each group, and 3 independent experiments were performed.

### 2.9. Detection of Mitochondria and Mitochondrial ROS Levels

The mitochondrial superoxide levels in C2C12 cells were detected by MitoSOX Red staining according to the manufacturer's protocol, and the fluorescence was analyzed by the ImageJ software. Twenty-four images were selected randomly and analyzed in each group. Four independent experiments were carried out.

### 2.10. Measurement of Serum CK-MM Isoenzyme Levels

Blood was collected from the tail vein of mice at 23 and 65 weeks of age. The samples were incubated at room temperature for 20 min to allow clotting and then centrifuged at 1000*g* for 20 min. The serum was collected and stored at −80°C until use. The serum level of the muscular isoform of creatine kinase (CK-MM) was measured in duplicate, using the CK-MM ELISA Kit (MBS705327, MyBioSource, San Diego, CA) according to the manufacturer's instructions.

### 2.11. Four-Limb Hanging Test

The four-limb hanging test was performed using mice at 37, 38, and 39 weeks of age. Mice were placed on a net, and then the net was inverted by hand. The hanging time was measured in 5 consecutive trials separated by 1 min intervals. Three independent experiments were performed, and the results are shown as the mean of 3 trials per group.

### 2.12. Rotarod Test

To assess motor coordination, *mdx* mice were tested on the rotarod (Ugo Basile, Mount Laurel, NJ) at 40 weeks of age. For training, mice were placed on the rotarod at 10 rpm for 5 min, on 3 consecutive days before the beginning of the experiment. The rotarod was accelerated from 10 to 50 rpm in 2 min, and the time at which the mouse fell off was recorded. Each mouse underwent 5 consecutive trials separated by 5 min intervals, and the results are shown as the mean values of 5 trials per group.

### 2.13. Data Analysis

Data are presented as means ± SEM. Statistical significance was determined using an unpaired Student's two-tailed *t*-test for 2 datasets. Differences between multiple groups were assessed by one-way analysis of variance (ANOVA) followed by the Tukey post hoc test. For all tests, *P* < 0.05 was considered statistically significant. All analyses were performed with the SigmaStat software (Systat Software Inc., San Jose, CA).

## 3. Results

### 3.1. Impaired Autophagy/Mitophagy and Increased ROS Levels in the Muscle of *mdx* Mice

**Figure 1:**
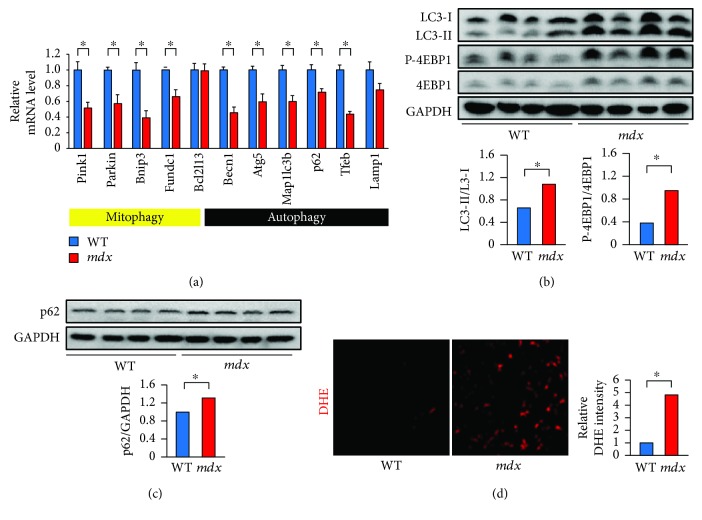
Downregulation of mitophagy and autophagy and increased ROS levels in the muscle of *mdx* mice. (a) Expression levels of mitophagy- and autophagy-related genes in the quadriceps muscle of WT and *mdx* mice at 22 weeks of age. Data were normalized to 18s ribosomal RNA. *n* = 4. (b) Representative Western blots for LC3, phosho-Ser65-4EBP1 (P-4EBP1), and total 4EBP1 in the quadriceps muscle (upper). Summary data of the LC3-II/LC3-I ratio and P-4EBP1 levels normalized to the total 4EBP1 level in the muscle (lower). *n* = 4. (c) Representative Western blots for p62 in the quadriceps muscle (upper) and summary data (lower). *n* = 4. (d) Representative dihydroethidium (DHE) staining in the quadriceps (left) and summary data of DHE fluorescence (right). Six randomly selected images were captured in each muscle section, and 4 mice were analyzed in each group. Scale bar: 50 *μ*m. ^∗^ *P* < 0.05. WT: wild type.

Impaired autophagic flux has been reported in the muscle of *mdx* mice [28]. We examined the mRNA levels of mitophagy- and autophagy-related genes in the quadriceps of 22-week-old *mdx* mice and compared them with those in age-matched wild type (WT) mice (Figure 1(a)). The mRNA expression levels of mitophagy-related genes, including *Pink1*, *parkin*, *Bnip3*, and *Fundc1*, were significantly reduced in the muscle of *mdx* mice. The expression levels of *Becn1*, *Atg5*, *Map1lc3b*, and *p62*, which are necessary for autophagy as well as mitophagy, were also lower in *mdx* than in WT mice (Figure 1(a)). The expression level of *transcription factor EB (Tfeb)*, a positive regulator of autophagy-related genes [18], in *mdx* mice was downregulated to less than half the level in control mice (Figure 1(a)).

To analyze the autophagic flux in the muscle of *mdx* mice, the protein levels of LC3-I, LC3-II, and p62 were monitored. LC3-I is processed to LC3-II during autophagosome formation, and then LC3-II is degraded after the autophagosome fuses with a lysosome. A low ratio of LC3-II to LC3-I levels (LC3-II/LC3-I) indicates an insufficiency in autophagosome formation, whereas a high LC3-II/LC3-I indicates insufficient autophagosome degradation or enhanced autophagosome formation. p62 is necessary for autophagy, and inhibiting autophagy increases the p62 protein level. As shown in Figure 1(b), the LC3-II/LC3-I was significantly higher in *mdx* than in WT mice. Although the mRNA levels of p62 were decreased in *mdx* mice (Figure 1(a)), the p62 protein levels were higher in *mdx* than in WT mice (Figure 1(c)). These results indicated that autophagy/mitophagy was suppressed in the muscle of *mdx* mice.

mTORC1, a major negative regulator of autophagy, is activated in *mdx* mice [28]. Since mTORC1 phosphorylates eukaryotic initiation factor 4E-binding protein 1 (4EBP1), we examined the phosphorylation levels of 4EBP1 (P-4EBP1). We found that the P-4EBP1 levels were significantly increased, suggesting that mTORC1 is activated, in the muscle of *mdx* mice (Figure 1(b)).

Suppressing mitophagy increases ROS levels [19]. To monitor ROS levels, sections of skeletal muscle were stained with dihydroethidium (DHE). Cellular superoxide converts DHE to ethidium bromide, which stains nuclear DNA with red fluorescence. The DHE fluorescence levels were much higher (4.8-fold) in *mdx* than in WT mice (Figure 1(d)). Together, these findings indicated that defects in autophagy/mitophagy could be involved in the increased ROS levels in the muscle of *mdx* mice.

### 3.2. Restoration of Autophagy/Mitophagy by Resveratrol in the Muscle of *mdx* Mice

**Figure 2:**
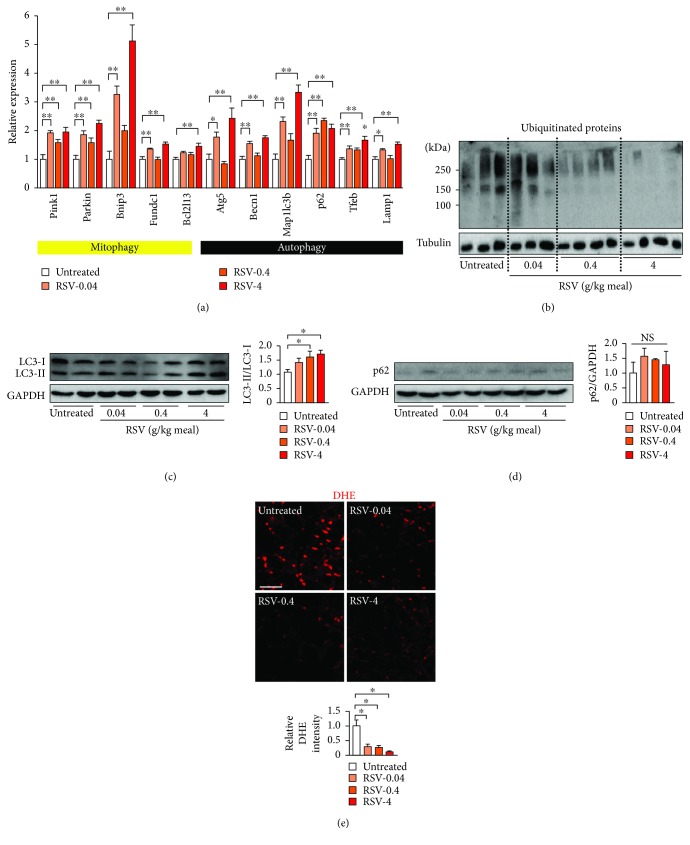
Effects of resveratrol on mitophagy, autophagy, and ROS levels in the muscle of *mdx* mice. (a) Expression levels of mitophagy- and autophagy-related genes in the quadriceps muscle from untreated *mdx* mice and *mdx* mice treated with resveratrol (RSV) at 0.04, 0.4, and 4 g/kg food (RSV-0.04, RSV-0.4, and RSV-4, respectively). *n* = 4 in each group. (b) Western blot analysis for ubiquitinated proteins in the quadriceps. (c) Representative Western blots for LC3 in the muscle tissue (left) and summary data of the LC3-II/LC3-I ratio (right). *n* = 4 in each group. (d) Representative Western blot for p62 in muscle (left) and summary data of p62 normalized to the GAPDH level (right). *n* = 4 in each group. (e) Representative dihydroethidium (DHE) staining in the quadriceps (upper) and summary data of DHE fluorescence intensity (lower). Six randomly selected images were captured in each muscle section, and 4 mice were analyzed in each group. Scale bar: 50 *μ*m. ^∗^ *P* < 0.05; ^∗∗^ *P* < 0.01. NS: not significant.

*mdx* mice were treated with 0.04, 0.4, or 4 g resveratrol/kg food, and the effect of resveratrol on autophagy/mitophagy was examined. The administration of resveratrol to *mdx* mice was started at 9 weeks of age, and the muscle tissues were examined at 65 weeks of age. During the experiment, two untreated *mdx* mice and 1, 1, and 2 mice receiving resveratrol at 0.04, 0.4, and 4 g/kg food, respectively, died of muscle tumors or unknown causes. No difference in the mean body weight was found among the untreated and resveratrol-treated mice at 34, 41, or 65 weeks of age (Table 1). The expression levels of mitophagy- and autophagy-related genes were significantly increased in the quadriceps of resveratrol-treated *mdx* mice compared with those of control *mdx* mice (Figure 2(a)). Approximately 2-fold increases in the *Pink1*, *parkin*, and *p62* mRNA levels were found in mice treated with all three doses of resveratrol in food. The *Bnip3*, *Fundc1*, *Atg5*, *Becn1*, *Map1lc3b*, *Tfeb*, and *Lamp1* levels were significantly upregulated by resveratrol at 0.04 and 4 g/kg food. The Bcl2l13 level was slightly but significantly elevated in the muscle of *mdx* mice treated with 4 g/kg food. Thus, resveratrol significantly increased the expression levels of mitophagy- and autophagy-related genes. In addition, resveratrol administered at 0.4 g/kg food significantly increased the SIRT1 mRNA levels by 2- to 3-fold in the quadriceps, diaphragm, and tibialis anterior (Supplemental Figure 1(a)).

An impairment in autophagic flux increases the ubiquitinated protein levels in tissues [19]. Western blot analysis showed that the administration of resveratrol dose-dependently decreased the levels of ubiquitinated proteins (Figure 2(b)), suggesting that resveratrol promoted the removal of ubiquitinated proteins from the muscle by inducing autophagic flux. Actually, we observed that resveratrol administration increased the LC3-II/LC3-I at 0.4 and 4 g/kg food (Figure 2(c)), but it did not significantly increase the p62 protein level (Figure 2(d)). These observations indicated that resveratrol increased the autophagic flux in *mdx* mice. The phosphorylation levels of 4EBP1 were not reduced by resveratrol (Supplemental Figure 1(b)), indicating that resveratrol did not inhibit the mTORC1 activity. DHE staining of muscular sections showed that resveratrol significantly decreased the ROS levels in the muscle of *mdx* mice. The ROS levels in the quadriceps of *mdx* mice treated with 0.04, 0.4, and 4 g/kg were 29, 26, and 11% of those in untreated *mdx* mice, respectively (Figure 2(e)).

### 3.3. Improvements in Skeletal Muscle Damage and Function in *mdx* Mice by Resveratrol

**Figure 3:**
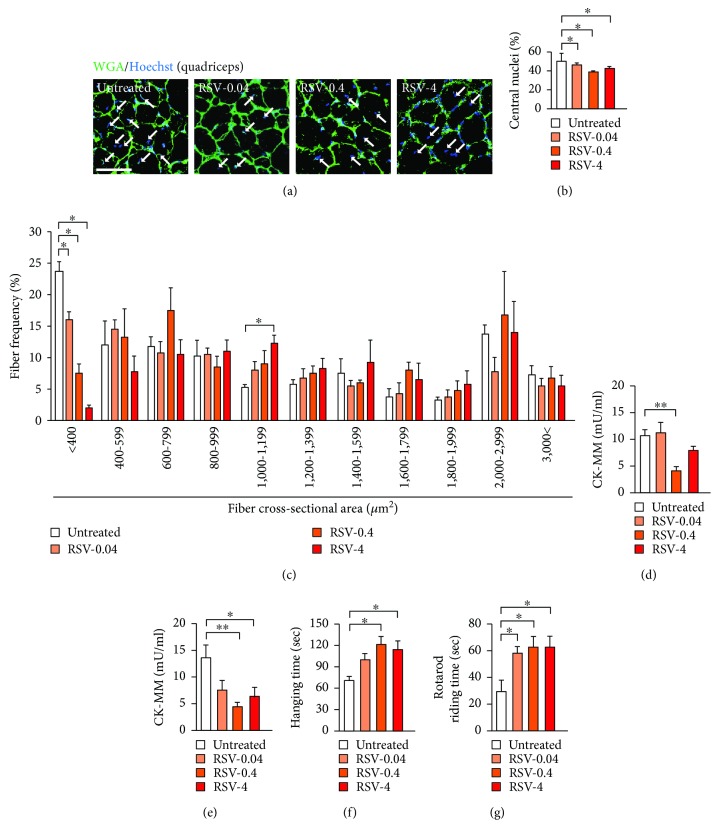
Resveratrol decreases muscular injury and improves muscle function in *mdx* mice. (a) Representative images of the quadriceps stained with FITC-conjugated wheat germ agglutinin (WGA, green) and Hoechst 33342 (blue) to detect myofiber membranes and nuclei, respectively. Muscle sections were obtained from untreated and resveratrol-treated *mdx* mice. Scale bar: 50 *μ*m. (b) Percentage of myofibers with central nuclei in *mdx* mice. *n* = 4 in each group. (c) Cross-sectional area of myofibers in the quadriceps muscles in *mdx* mice. *n* = 4 mice per group. (d, e) Serum levels of the muscle isoform of creatine kinase (CK-MM) in the *mdx* mice at 23 (d) and 65 weeks old (e). *n* = 5–6 mice per group. (f) Hanging time assessed by the inverted hang test of *mdx* mice at 37 weeks of age. (g) Rotarod riding time in *mdx* mice at 40 weeks of age. *n* = 4–5 mice per group. ^∗^ *P* < 0.05; ^∗∗^ *P* < 0.01.

The autophagy/mitophagy restoration and ROS reduction by resveratrol may affect muscle degeneration and regeneration. Since regenerating myofibers contain central nuclei, sections of the quadriceps from *mdx* mice at 65 weeks of age were treated with Hoechst 33342 and FITC-conjugated WGA to stain nuclei and plasma membranes, respectively, and examined by confocal microscopy (Figures 3(a) and 3(b)). Resveratrol treatment at all three doses significantly decreased the number of myofibers with central nuclei, showing that resveratrol reduced the number of newly generated myofibers. Analysis of the cross-sectional areas of myofibers revealed that resveratrol dose-dependently decreased the number of fine fibers (under 400 *μ*m^2^ in cross-sectional area) compared with the number in untreated *mdx* mice (Figure 3(c)). In contrast, the number of wider myofibers (1000 to 1199 *μ*m^2^ in cross-sectional area) was significantly increased by resveratrol administered at 4 g/kg food (Figure 3(c)). These results suggested that resveratrol decelerated the turnover rates and promoted the maturation of myofibers in *mdx* mice.

Whether resveratrol affected number of satellite cells, mRNA levels of Pax 7, a marker of satellite cells, were measured in the quadriceps and soleus of *mdx* mice. However, Pax7 mRNA levels were not affected by resveratrol administration (Supplemental Figure 2). Recently, AMPK activation in satellite cells has been shown to inhibit apoptosis and promote muscle repair [29]. To detect AMPK activation, phosphorylation levels of AMPK in the quadriceps were examined, but we could not detect significant increase of activated AMPK levels by resveratrol (Supplemental Figure 3).

The serum levels of CK-MM reflect skeletal muscle cell damage. Thus, to examine whether muscle injuries were attenuated by resveratrol, the serum CK-MM levels were examined. Resveratrol administered at 0.4 g/kg food to *mdx* mice significantly decreased the CK-MM levels to about one-third of those in untreated *mdx* mice at 23 and 65 weeks of age (Figures 3(d) and 3(e)). At 65 weeks of age, the administration of resveratrol at 4 g/kg food also significantly decreased the CK-MM levels, which were less than half those observed in untreated *mdx* mice (Figure 3(e)).

To investigate whether resveratrol improves skeletal muscle motor function, *mdx* mice were examined by the inverted hang test and the rotarod test, which reflect fatigue resistance and muscular coordination. At 37 weeks of age, the average hanging time in untreated *mdx* mice was 69 sec, and resveratrol treatment at 0.4 and 4 g/kg food significantly extended the time to 121 sec and 114 sec, respectively. At 40 weeks of age, an approximate 2-fold extension of riding time on the rotating rod was observed in *mdx* mice treated with resveratrol as low as 0.04 g/kg food (Figure 3(g)). Resveratrol at 0.4 g/kg and 4 g/kg food extended the riding time on the rotarod to durations similar to those seen with 0.04 g/kg food (Figure 3(g)). Therefore, treatment with resveratrol, especially at a dose of 0.4 g/kg food, decreased the muscle injury and improved physical activities of *mdx* mice.

### 3.4. Mitophagy Induction by Resveratrol in C2C12 Cells

**Figure 4:**
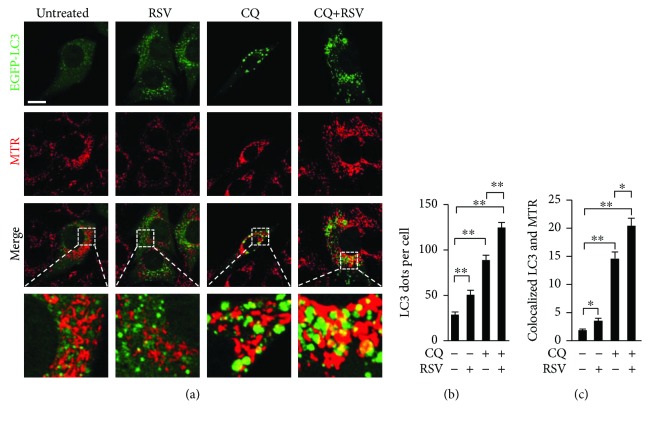
Resveratrol induces mitophagy in C2C12 cells. (a) Representative images of EGFP-LC3 and MitoTracker Red (MTR) and the merged images in C2C12 cells. Cells were cultured in the absence or presence of resveratrol (RSV, 30 *μ*M), chloroquine (CQ, 50 *μ*M), or RSV and CQ together, for 6 h. Yellow indicates EGFP-LC3 dots (green) colocalized with mitochondria (red). Scale bar: 10 *μ*m. (b) Summary data of the number of LC3 dots per cell. (c) Summary data of the number of EGFP-LC3 dots colocalized with fragmented mitochondria per cell. In (c) and (d), the data were obtained from 40 randomly selected cells from 4 independent experiments. ^∗^ *P* < 0.05; ^∗∗^ *P* < 0.01.

Resveratrol induces autophagy [15, 16]. To examine whether resveratrol also promotes mitophagy, we examined the effect of resveratrol on mitophagy using C2C12 myoblast cells. Autophagosome formation can be monitored by the appearance of EGFP-LC3 dots in cells [30]. EGFP-LC3 was expressed in C2C12 cells, and the number of EGFP-LC3 dots was counted. Treating the cells with resveratrol significantly increased the number of EGFP-LC3 dots compared with control cells (Figures 4(a) and 4(b)). Treating the cells with chloroquine (CQ), which suppresses lysosome function thereby inhibiting the degradation of autophagosomes, increased the number of LC3 dots (Figures 4(a) and 4(b)). In the presence of CQ, resveratrol treatment further increased the number of LC3 dots (Figures 4(a) and 4(b)). These observations indicated that resveratrol enhanced autophagosome formation.

To examine whether mitophagy was accelerated by resveratrol, we stained mitochondria with MitoTracker Red (MTR) and examined the colocalization of mitochondria with EGFP-LC3 dots. While there were few mitochondria-containing autophagosomes in the control cells, the administration of CQ significantly increased the number of EGFP-LC3 dots colocalized with mitochondria (Figure 4(a)). Thus, in the absence of CQ, mitochondria were constantly degraded by mitophagy in C2C12 cells (Figures 4(a) and 4(c)). Resveratrol significantly increased the number of EGFP-LC3 dots colocalized with mitochondria in the absence of CQ (Figures 4(a) and 4(c)). The highest numbers of EGFP-LC3 dots colocalized with mitochondria were detected in cells treated with resveratrol and CQ (Figures 4(a) and 4(c)). These results indicated that resveratrol promoted both autophagy and mitophagy.

### 3.5. Mitochondrial ROS Reduction by Resveratrol-Induced Mitophagy

**Figure 5:**
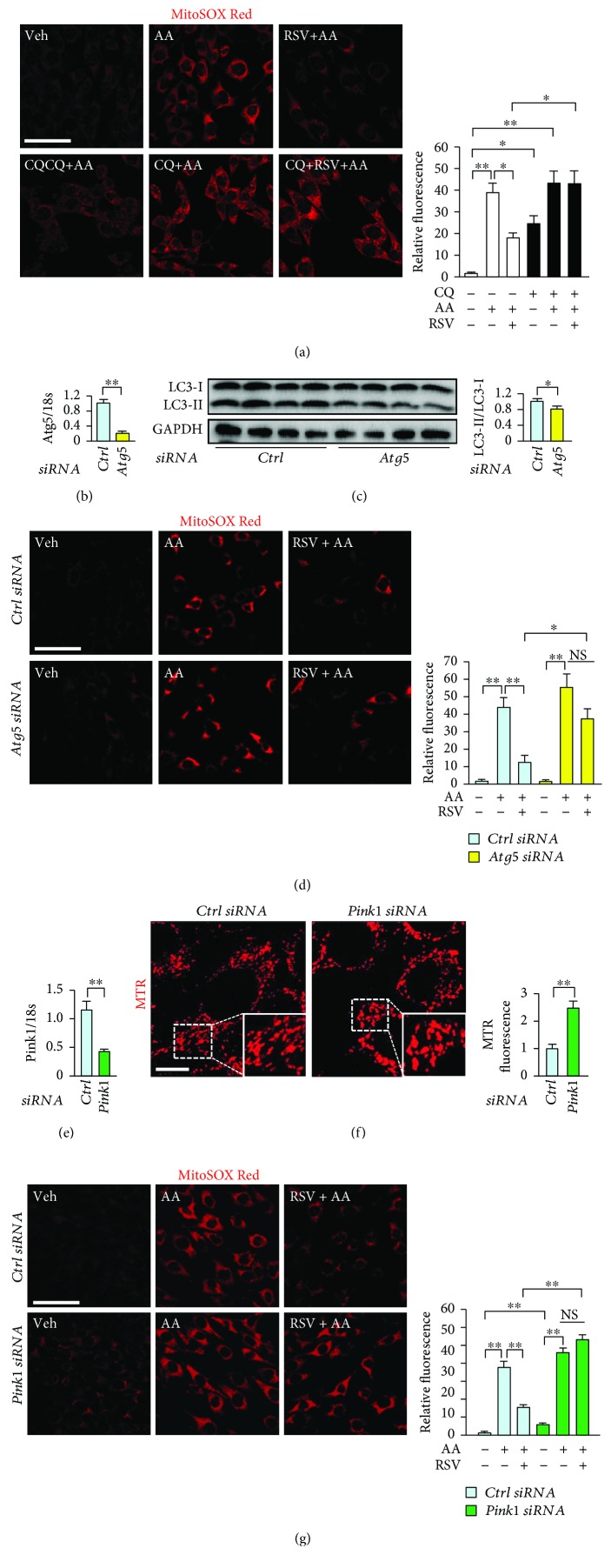
Resveratrol decreases ROS levels via mitophagy in C2C12 cells. (a) Representative images of MitoSOX Red fluorescence in C2C12 cells. Cells were cultured with vehicle (Veh), antimycin A (AA, 10 *μ*M), or AA and resveratrol (RSV, 30 *μ*M) in the presence or absence of chloroquine (CQ, 50 *μ*M) for 24 h (left). Summary data of MitoSOX Red fluorescence intensity per cell (right). *n* = 6. Scale bar: 50 *μ*m. (b) mRNA level of Atg5 normalized to 18s ribosomal RNA in C2C12 cells transfected with *control (Ctrl) siRNA* or *siRNA* against mouse *Atg5*. *n* = 4. (c) Representative Western blot for LC3 in cells transfected with control or *Atg5 siRNA* in C2C12 cells. *n* = 4. (d) Representative images of MitoSOX Red fluorescence in C2C12 cells transfected with control or *Atg5 siRNA* and treated with vehicle, AA, or AA and resveratrol for 24 h (left). Scale bar: 50 *μ*m. Summary data of MitoSOX Red fluorescence intensity (right). *n* = 6. (e) Level of *Pink1* mRNA in cells treated with control or *Pink1 siRNA* in C2C12 cells. *n* = 4. (f) Representative images of MitoTracker Red (MTR) fluorescence in C2C12 cells transfected with control or *Pink1 siRNA* (left). Scale bar: 10 *μ*m. Summary data of MitoTracker Red fluorescence intensity (right). *n* = 4. (g) Representative images of MitoSOX Red fluorescence in C2C12 cells transfected with control or *Pink1 siRNA* and treated with vehicle, AA, or AA and resveratrol for 24 h (left). Scale bar: 50 *μ*m. Summary data of MitoSOX Red fluorescence intensity per cell (right). *n* = 6. ^∗^ *P* < 0.05; ^∗∗^ *P* < 0.01. NS: not significant.

Antimycin A (AA), an inhibitor of the electron transport chain in mitochondria, depolarizes mitochondria and increases the mitochondrial ROS levels. C2C12 cells were treated with AA, and the mitochondrial ROS levels were monitored with MitoSOX Red, a fluorescent indicator of mitochondrial superoxide levels. Treating the cells with AA increased the mitochondrial ROS levels, and this increase was significantly suppressed by resveratrol (Figure 5(a)). The addition of CQ completely cancelled this effect of resveratrol on the ROS levels (Figure 5(a)), indicating that the autophagic flux was required for resveratrol's antioxidative effect.

Atg5, an E3 ubiquitin-like ligase, is involved in autophagic vesicle formation. Because Atg5 is deacetylated by SIRT1 [14], we examined the effect of Atg5 knockdown on resveratrol's function. Treating the C2C12 cells with *Atg5-siRNA* decreased the LC3-II/LC3-I ratio, indicating that the *Atg5-siRNA* inhibited autophagy (Figures 5(b) and 5(c)). MitoSOX Red staining showed that resveratrol failed to decrease the AA-induced ROS levels in cells treated with *Atg5-siRNA* (Figure 5(d)). Thus, disrupting autophagy/mitophagy with *Atg5-siRNA* inhibited resveratrol's antioxidative function.

Pink1 is indispensable for mitophagy [31]. Therefore, to inhibit mitophagy, C2C12 cells were treated with *Pink1-siRNA* (Figures 5(e)–5(g)). In the absence of AA, the knockdown of Pink1 alone altered the mitochondrial morphology and increased the size and area of mitochondria in C2C12 cells (Figures 5(e) and 5(f)), indicating that mitophagy was disrupted by the *Pink1-siRNA*. MitoSox Red staining showed that *Pink1-siRNA* completely cancelled resveratrol's antioxidative function against AA (Figure 5(g)). These findings together indicated that the induction of mitophagy by resveratrol reduced the number of damaged mitochondria and decreased the ROS levels.

## 4. Discussion

Membrane fragility due to dystrophin deficiency causes intracellular Ca^2+^ dysregulation, resulting in mitochondrial dysfunction and ROS production [32]. Damaged mitochondria are a major source of cellular ROS and are selectively degraded by mitophagy, which decreases cellular ROS levels [17–19]. We showed that resveratrol induced mitophagy (Figure 4) and reduced the ROS levels in C2C12 cells in a mitophagy-dependent manner (Figure 5). *Pink1-siRNA* alone significantly increased the ROS levels in the absence of AA, indicating that mitophagy continuously contributes to the decrease in cellular ROS levels (Figure 5(g)). Although the knockdown efficiency by *Atg5-siRNA* was greater than that by *Pink1-siRNA* (Figures 5(b) and 5(e)), *Atg5-siRNA* alone could not increase the ROS levels in the absence of AA (Figure 5(d)). Atg5 is dispensable for the mitophagy occurring during erythroid maturation, and Atg5-independent mitophagy is found in various organs [33]. Thus, an Atg5-independent mitophagy pathway may contribute to decrease the ROS levels in C2C12 cells.

The expression of autophagy-related genes, i.e., *Atg12*, *Map1lc3b*, *Gabarapl1*, and *Bnip3*, was previously shown to be suppressed in *mdx* mice [28]. In this study, we found that other autophagy/mitophagy-related genes, i.e., *Pink1*, *parkin*, *Fundc1*, *Becn1*, *Atg5*, *p62*, and *Tfeb*, were downregulated in the quadriceps of *mdx* mice (Figure 1). TFEB is a master transcription factor for autophagy and lysosomal biogenesis [18]. Because the *Tfeb* mRNA levels were downregulated in *mdx* mice (Figure 1(a)), a decrease in TFEB level may downregulate autophagy/mitophagy-related genes in *mdx* mice. In addition, increase in the P-4EBP1 levels suggested the activation of mTORC1 in *mdx* mice (Figure 1(b)). mTORC1 is reported to downregulate autophagy-related genes in animal models of muscular dystrophies and DMD patients [28, 34]. Consistent with this finding, inhibiting mTORC1 by administering a low-protein diet or rapamycin ameliorates dystrophic muscle phenotypes [28, 34]. Since TFEB is phosphorylated and excluded from the nucleus by mTORC1, TFEB's inactivation by mTORC1 may also contribute to the downregulation of autophagy/mitophagy-related genes. We found that resveratrol restored the expression levels of autophagy/mitophagy machineries and autophagic flux (Figures 2(a)–2(c)). However, the phosphorylation levels of 4EBP1 were not changed by resveratrol (Supplemental Figure 1(b)), indicating that resveratrol did not affect the mTORC1 activity. FOXOs are known to positively regulate autophagy/mitophagy-related genes [19]. We previously showed that resveratrol decreases ROS levels in C2C12 cells by promoting the activation of FOXOs [35]. Because the knockdown of *Foxos*, i.e., *Foxo1*, *Foxo3a*, and *Foxo4*, by their *siRNAs* completely inhibited resveratrol's antioxidative function in AA-treated C2C12 cells [35], the activation of FOXOs by resveratrol may upregulate the autophagy/mitophagy-related genes and facilitate the autophagy/mitophagy flux in *mdx* mice. In addition, the upregulation of *Sirt1* mRNA by resveratrol in *mdx* mice (Supplemental Figure 1(a)) may have been caused by the activation of FOXOs, since FOXOs also induce *Sirt1* mRNA [4].

*SIRT1 siRNA* also inhibits resveratrol's antioxidative function in AA-treated C2C12 cells [35], suggesting that resveratrol activates FOXOs via SIRT1 activation in *mdx* mice. In addition, the deacetylation and activation of Atg5, Atg7, and LC3 by SIRT1 could be involved in the increased autophagy/mitophagy flux caused by resveratrol.

Recently, mitochondrial dysfunction and mitophagy insufficiency were shown to be involved in the pathogenesis of progeroid syndromes. Mitophagy disturbance worsens the phenotypes of xeroderma pigmentosum group A (XPA) deficiency and ataxia telangiectasia (AT), both of which are DNA repair disorders [36, 37]. DNA repair failure activates poly (ADP-ribose) polymerase 1 (PARP1), and then NAD^+^ is depleted by the activated PARP1, thereby decreasing the activity of the NAD^+^-dependent deacetylase SIRT1. Increased NAD^+^ levels activate SIRT1's activity, and indeed, activating SIRT1 by adding nicotinamide riboside, an NAD^+^ precursor, improves the mitochondria quality via mitophagy induction and retards the progression of the DNA repair disorders [36, 37]. Importantly, *mdx* mice have been shown to have increased PARP activities and low NAD^+^ levels in their muscle tissues [38]. Because PARP is activated by DNA damage, *mdx* mice are expected to have enhanced levels of DNA damage, which may be derived from the disturbance of mitophagy. Replenishing the NAD^+^ by administering nicotinamide riboside reduced the nuclear ADP-ribosylated protein levels and improved the muscle function and heart pathology in *mdx* mice [38]. Similar to XPA and AT, nicotinamide riboside may induce mitophagy and ameliorate phenotypes of dystrophin-deficient mice. Since nicotinamide riboside is much more costly than resveratrol, resveratrol has an economic advantage over nicotinamide riboside to treat DMD.

Stem cell depletion also plays a role in the progression of muscular dystrophies [3, 39]. SIRT1 induces the proliferation of myoblast cells, and muscle-specific SIRT1 knockout mice exhibit impaired muscle regeneration [40]. Because resveratrol appeared to decelerate the muscular turnover rate and to suppress excess muscle regeneration (Figures 3(a)–3(c)), resveratrol may preserve the number of muscle stem cells in DMD. However, resveratrol administration to *mdx* mice did not increase Pax7 expression levels (Supplemental Figure 2). Thus, the resveratrol's main function on the muscle of *mdx* mice seems to inhibit cell death and promote maturation of muscle cells by reducing oxidative stress.

In this study, we administered resveratrol to *mdx* mice at three doses to determine its optimal dose. The most effective dose of resveratrol was 0.4 g/kg food for muscle injury, function, and autophagic activity, although the administration of resveratrol at 0.04 g/kg or 4 g/kg was also effective. Because resveratrol is rather hydrophobic, it may accumulate in lipids such as cellular membranes and adipose tissues. For its clinical evaluation, the optimal dosage of resveratrol for treating muscular dystrophies needs to be determined. Our findings indicate that resveratrol would be effective for muscular dystrophy patients and may provide a combination therapy with other medicines such as glucocorticoids.

## Figures and Tables

**Table 1:** Effect of resveratrol on body weight in *mdx* mice.

Age (weeks)	Dose of resveratrol (g/kg food)			
	0	0.04	0.4	4
	Body weight (g)			
34	34 ± 1	33 ± 0	32 ± 1	34 ± 1
41	35 ± 1	34 ± 0	33 ± 2	34 ± 1
65	33 ± 2	31 ± 0	30 ± 1	31 ± 1

No significant difference was observed in the body weight of *mdx* mice treated with 0, 0.04, 0.4, or 4 g resveratrol/kg food at any age.

## Data Availability

The data used to support the findings of this study are available from the corresponding author upon request.

## Abbreviations

AA: Antimycin A
CK-MM: Muscular isoform of creatine kinase
CQ: Chloroquine
DHE: Dihydroethidium
DMD: Duchenne muscular dystrophy
4EBP1: Eukaryotic initiation factor 4E-binding protein 1
FOXOs: Forkhead box O transcription factors
Mitophagy: Autophagy of damaged mitochondria
mTORC1: Mechanistic target of rapamycin complex 1
MTR: MitoTracker Red
PGC-1*α*: Peroxisome proliferator-activated receptor gamma coactivator 1-alpha
Pink1: PTEN-induced putative kinase 1
ROS: Reactive oxygen species
RSV: Resveratrol
SOD2: Superoxide dismutase 2
WGA: Wheat germ agglutinin
WT: Wild type.

## Supplementary Materials

Supplementary Materials Supplemental Figure 1: effects of resveratrol on SIRT1 expression and P-4EBP1 levels of muscles from mdx mice. (a) SIRT1 mRNA levels analyzed by a qPCR method in the quadriceps, diaphragm, and tibialis anterior (TA) muscles from untreated and resveratrol- (RSV-) treated mdx mice. *n* = 4. (b) Representative Western blots (upper) and summary data (lower) for P-4EBP1 and total 4EBP1 in muscles from mdx mice. *n* = 4. ^∗^ *P* < 0.05, NS: not significant. Supplemental Figure 2: effects of resveratrol on Pax7 mRNA expression levels of muscles from mdx mice. Pax7 mRNA levels analyzed by a qPCR method in the quadriceps and soleus muscles from untreated and resveratrol- (RSV-) treated mdx mice. *n* = 4. NS: not significant. Supplemental Figure 3: effects of resveratrol on P-AMPK and AMPK levels of quadriceps muscles from mdx mice. Western blots (upper) and summary data (lower) for P-AMPK and AMPK in muscles from mdx mice. *n* = 4. NS: not significant. Supplemental Table 1: primer sequences for quantitative PCR. Supplemental Table 2: antibodies used in the present study.
