# Supplementary material for: Resveratrol Decreases Oxidative Stress by Restoring Mitophagy and Improves the Pathophysiology of Dystrophin-Deficient mdx Mice
Source: Oxid Med Cell Longev. 2018 Oct 29;2018:9179270. doi: 10.1155/2018/9179270 (PMC6231358; doi:10.1155/2018/9179270)
Supplement: Supplementary Materials — Supplemental Figure 1: effects of resveratrol on SIRT1 expression and P-4EBP1 levels of muscles from mdx mice. (a) SIRT1 mRNA levels analyzed by a qPCR method in the quadriceps, diaphragm, and tibialis anterior (TA) muscles from untreated and resveratrol- (RSV-) treated mdx mice. n = 4. (b) Representative Western blots (upper) and summary data (lower) for P-4EBP1 and total 4EBP1 in muscles from mdx mice. n = 4. ∗ P < 0.05, NS: not significant. Supplemental Figure 2: effects of resveratrol on Pax7 mRNA expression levels of muscles from mdx mice. Pax7 mRNA levels analyzed by a qPCR method in the quadriceps and soleus muscles from untreated and resveratrol- (RSV-) treated mdx mice. n = 4. NS: not significant. Supplemental Figure 3: effects of resveratrol on P-AMPK and AMPK levels of quadriceps muscles from mdx mice. Western blots (upper) and summary data (lower) for P-AMPK and AMPK in muscles from mdx mice. n = 4. NS: not significant. Supplemental Table 1: primer sequences for quantitative PCR. Supplemental Table 2: antibodies used in the present study. [file 9179270.f1.pdf]

Supplemental data for Sebori *et al.*

Supplemental Figure 1.

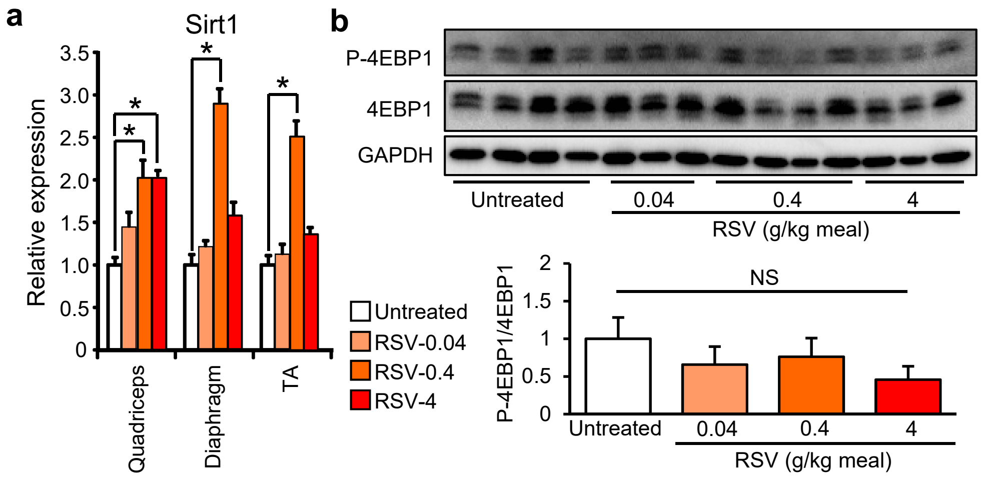

Supplemental Figure 2.

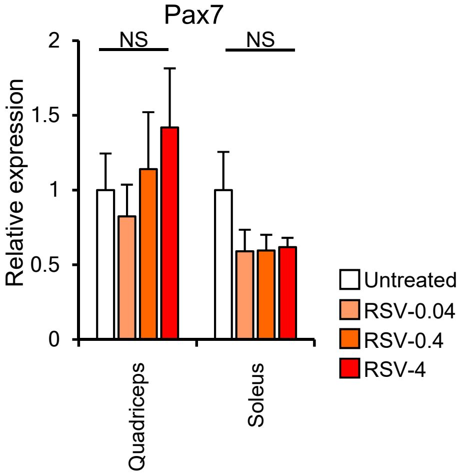

Supplemental Figure 3.

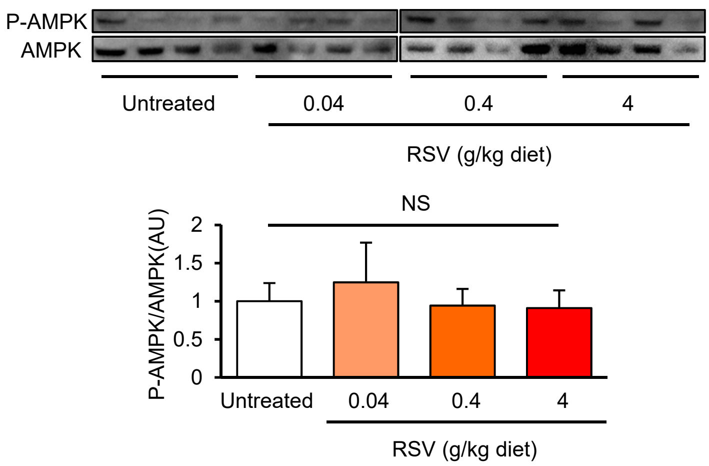

Supplemental Table 1. Primer sequences for quantative PCR.

Gene	Forward	Reverse
**Map1lc3b**	5'-CGTCCTGGACAAGACCAAGT-3'	5'-ATTGCTGTCCCGAATGTCTC-3'
**Bnip3**	5'-TCCACTAGCACCTTCTGATGA-3'	5'-GAACACCGCATTTACAGAACAA-3'
**Atg5**	5'-TTGGAACATCACAGTACATTT-3'	5'-AACGAAATCCATTTTCTTCTG-3'
**Becn1**	5'-CTGACAGACAAATCTAAGGAG	5'-AATAGGAGCCGCCACTGCCTC-3'
**p62**	5'-CCTTGCCCTACAGCTGAGTC-3'	5'-CACACTCTCCCCCACATTCT-3'
**Pink1**	5'-TGAGGAGCAGACTCCCAGTT-3'	5'-AGTCCCACTCCACAAGGATG-3'
**Parkin**	5'-TGGAAAGCTCCGAGTTCAGT-3'	5'-CCTTGTCTGAGGTTGGGTGT-3'
**Lamp1**	5'-ACATCAGCCCAAATGACACA-3'	5'-GGCTAGAGCTGGCATTCATC-3'
**Tfeb**	5'-AACAGTGCTCCCAACAGTCC-3'	5'-GGCGCATAATGTTGTCAATG-3'
**Fundc1**	5'-CCCCCTCCCCAAGACTATGAA-3'	5'-CCACCCATTACAATCTGAGTAGC-3'
**Bcl2l13**	5'-CTCAGCCAGCAGTGACATA-3'	5'-GGCACCAAAAGCTTATTCCA-3'
**18s**	5'-CGGACAGGATTGACAGATTG-3'	5'-CAAATCGCTCCACCAACTAA-3'
**Sirt1**	5'-GACGCTGTGGCAGATTGTTA-3'	5'-GGAATCCCACAGGAGACAGA-3'

Supplemental Table 2. Antibodies used in the present study.

Antibody	Source	Catalog number
anti-LC3AB	Cell Signaling Technology	12741
anti-phosho-Ser65-4EBP1	Cell Signaling Technology	9451
anti-total 4EBP1	Cell Signaling Technology	9452
anti-ubiquitin	Cell Signaling Technology	3936
anti-Phospho-AMPKα (Thr172) (40H9)	Cell Signaling Technology	2535
anti-AMPKα Antibody	Cell Signaling Technology	2532
anti-p62	Progen	GP62-C
anti-GAPDH	Sigma-Aldrich	G8795
anti-α-tubulin	Sigma-Aldrich	T5168
